# A unified model of the biology of peripartum depression

**DOI:** 10.1038/s41398-023-02439-w

**Published:** 2023-04-28

**Authors:** Gal Levin, Tsachi Ein-Dor

**Affiliations:** Reichman University. Please address all correspondence to: Tsachi Ein-Dor, Baruch Ivcher School of Psychology, Reichman University, University St. 8, Herzliya, 4610101 Israel

**Keywords:** Pathogenesis, Prognostic markers

## Abstract

Peripartum depression (PPD) is a prevalent and debilitating disorder that adversely affects the development of mothers and infants. Recently, there has been a plea for increased mental health screening during the peripartum period; however, currently, there is no accurate screening tool to identify women at risk of PPD. In addition, some women do not respond to current treatment schemes and develop treatment-resistant depression. The current perspective aims to propose a unified understanding of the biological underpinnings of PPD (UmPPD) that considers the heterogeneity in the onset, symptoms cluster, and severity of PPD. Such a model could promote basic and applied research on PPD and suggest new treatment avenues. The central hub of the model is the kynurenine pathway (KP) and the KP-serotonin ratio. The forces and specific processes at play that cause an imbalance within the KP and between KP and serotonin are inflammation, stress, reproductive hormones (especially estradiol and progesterone), and oxytocin. UmPPD predicts that the most severe PPD would comprise prolonged inflammation, ongoing or multiple stressors, excessive estrogen, progesterone resistance, and avoidance of breastfeeding, skin-to-skin contact, and social proximity. These factors would be associated with a higher likelihood of developing PPD, early onset, and more significant symptom severity. In addition, subtypes of PPD would consist of different compositions and expressions of these components, with one central common factor. UmPPD could aid in directing future research and possibly detecting critical processes that could help discover, develop, and utilize novel treatments for PPD.

## A unified model of the biology of peripartum depression

Peripartum depression (PPD) is a severe psychiatric disorder that is understudied (both clinically and experimentally) and underdiagnosed, with an onset during pregnancy or within the first four weeks postpartum [1]. PPD negatively impacts the mother, with suicide accounting for approximately 20% of postpartum deaths [2], while adversely affecting infants’ behavioral, emotional, and cognitive development [3]. Estimated incidences of PPD range from 13% to 19% [4]. Given that approximately 920 million women give birth each year, this would translate to as many as 174 million affected women worldwide suffering from minor or major depression, making it one of the most frequent medical complications surrounding childbirth. In addition, research estimates that approximately 50% of women with PPD go undiagnosed [5], and that the financial tool of untreated perinatal mood disorders staggers to an average of 31,800$ per affected mother-child dyad [6]. Accordingly, there has been a plea for increased mental health screening during the peripartum period [7], yet currently, there is no accurate screening tool for identifying women at risk of PPD [8].

On top of that, a recent study revealed that at least 5% of women with PPD develop treatment-resistant depression (TRD) within one year after the diagnosis of PPD – i.e., did not respond to at least three different antidepressant treatments or one antidepressant and one antipsychotic treatment within a period of one year [9]. There is, however, increasing evidence that the rates of treatment-resistant depression might be considerably higher [10]. In addition, although a recent meta-analysis indicated that psychological treatments of PPD are effective, this effectiveness is only moderate in effect size (adjusted g = 0.53) and has high variability [11]. Therefore, there is also an urgent need to develop effective treatment strategies and an adequate medical approach to PPD. Unfortunately, the heterogeneity of PPD and the lack of biomarkers to create more homogenous clinical groups make it very difficult to find optimal treatment approaches for PPD. Here, we intend to begin to fill in the gaps in research by suggesting a unified model for the biological underpinning of PPD; such a model might promote the detection of novel potential targets for the future development of effecting treatment schemes and account for the heterogeneity in likelihood, onset, and severity of PPD.

## PPD

In the Diagnostic and Statistical Manual of Mental Disorders, Fifth Edition (DSM-5), PPD is classified as “Major Depression Disorder, with peripartum onset,” given that symptoms manifestation begins during pregnancy in about a third of women with PPD [12]. The diagnosis requires the presence of 5 or more symptoms, including depressed mood, loss of interest or pleasure in activities once enjoyed, changes in weight or appetite, trouble sleeping or sleeping too much, fatigue, diminished ability to think or concentrate, feeling worthless or guilty, change in locomotion, and thinking about death or suicide. The need to possess only 5 symptoms to be classified as having PPD implies that PPD is a family of interconnected disorders, not one cohesive and fixed disorder. In addition to the symptoms that share equivalence with major depressive disorder (MDD), some symptoms are unique to PPD, including a lack of interest in the baby, not feeling bonded to the baby, feeling very anxious about or around the baby, feelings of being a bad mother, and/or fear of harming the baby or oneself. The peripartum specifier states that the onset of symptoms ought to occur during pregnancy or four weeks postpartum. However, it has been recommended that the diagnostic criteria be expanded from 4 weeks to 6 months after delivery [4].

## Toward a unified understanding of PPD

**Fig. 1:**
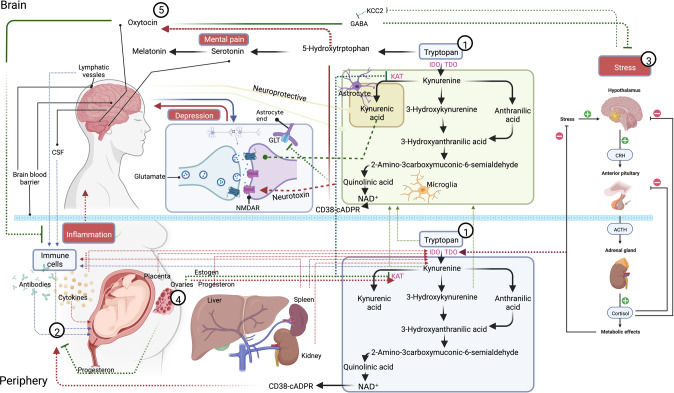
1. Tryptophan is mainly synthesized into serotonin (<5%) or kynurenine and its breakdown products (>95%), culminating in the generation of nicotinamide adenine dinucleotide (NAD+), an important cellular energy source. The kynurenine pathway has two branches – one that leads to quinolinic acid and NAD+, which comprises several neurotoxins, and one that leads to kynurenic acid, which is neuroprotective. 2. Immune cells, in different phases of pregnancy, intensify kynurenine production at the expense of serotonin, mainly via the indoleamine 2,3-dioxygenase (IDO) enzyme. 3. Stress further intensifies this process, chiefly via the effect of the glucocorticoid receptor on the tryptophan dioxygenase (TDO) enzyme. 4. Reproductive hormones, especially estradiol, intensify the production of quinolinic acid and NAD+ at the expense of kynurenic acid via the inhibition of kynurenine aminotransferase (KAT). Progesterone is a protective hormone, but the body may develop progesterone resistance because of the immune system response. Quinolinic acid (QUIN), which is excessively metabolized under these conditions, is a potent agonist of glutamate’s N-methyl-D-aspartate receptor (NMDAR), which exerts neurotoxic effects via at least nine different mechanisms and brings about the main symptoms of depression. QUIN also inhibits glutamate uptake into astrocytes, inducing cessation of responding and anhedonia. Serotonin and melatonin are depleted, affecting appetite, learning, memory, and sleep. It also hinders one of the main pathways of pain reduction – that of serotonin – that could promote mental pain and suicide ideation 5. CD38 generates cADPR out of NAD + that further activates the immune response and secretion of oxytocin (OT). OT can then inhibit the immune and stress responses, indirectly reducing overall symptoms. Lack of physical touch, breastfeeding, and support reduce OT levels, and prolonged inflammation may hinder the secretion of OT via CD38-cADPR. Chronic stress may also downregulate the expression of potassium-chloride cotransporter-2 (KCC2), causing OT to exacerbate the stress response rather than inhibit it because of the dysregulation of gamma-aminobutyric acid receptors (GABA_A_). The figure was created with BioRender.com.

Any theory on the biological underpinning of PPD needs to be able to explain the entire constellation of symptoms and the individual differences in their manifestation. To date, research has suggested many players in PPD, the main ones being reproductive hormones [13], stress hormones, particularly those of the Hypothalamic-Pituitary-Adrenal (HPA) axis [14], inflammatory responses [15], the pathways of monoamine neurotransmitters, especially serotonin [16], neurotrophins [17], and endocrine hormones, particularly oxytocin [18]. In addition, research in various fields (e.g., psychology [19], genetics [20], epigenetic [21], endocrinology [22]) has indicated that events in the internal and/or external environment, chiefly stress-related, as well as genetic polymorphism, can modulate the effects of all leading players on the development and maintenance of PPD. Here, we propose a unified model for PPD (UmPPD) that may better guide the search for potent biomarkers (see Fig. 1), which is based on state-of-the-art research and theoretical reasoning (e.g., [23, 24]).

## The unified model of PPD (UmPPD)

### Tryptophan, the kynurenine pathway, and serotonin

A central player in the UmPPD is the α-amino acid, tryptophan. It is metabolized into several bioactive molecules, out of which two are central to PPD: serotonin (<5% of tryptophan) and kynurenine (>95%) and its breakdown products, as part of the kynurenine metabolic pathway [24] (KP). In the brain and immune cells, tryptophan is converted into kynurenine by the indoleamine 2,3-dioxygenase (IDO) enzyme and, in many other tissues, by the tryptophan dioxygenase (TDO) [25] enzyme. The greater the expression of IDO and/or TDO, the greater the conversion of tryptophan into kynurenine at the expense of serotonin. Sufficient serotonin is crucial for sleep regulation (as serotonin is the precursor of melatonin) [26], weight and appetite [27] maintenance, and one of the climacteric symptoms of PPD – reduction of pain [28]. Although physical and mental pain only partially overlap [29], research has noted that serotonin also plays a vital role in mental pain inhibition [30]. In ideal, everyday conditions, the body maintains a delicate balance between the production of kynurenine and serotonin.

In addition, the kynurenine pathway has two main branches: kynurenine may be converted into 3-hydroxykynurenine (3HK) followed by 3-hydroxyanthranilic acid (3HAA), quinolinic acid (QUIN), and ultimately nicotinamide adenine dinucleotide (NAD^+^) [31], with NAD^+^ being an essential cellular energy source [32]. However, by itself, QUIN is a neuroactive agonist of glutamate’s N-methyl-D-aspartate receptor (NMDAR) that incurs neurotoxic effects via at least nine processes (reviewed in [33]) such as generation of reactive oxygen species (ROS), disruption of the blood-brain barrier, and destabilization of the cellular cytoskeleton; 3HK and 3HAA are also neurotoxic by contributing to the generation of free radicals [34] leading to lipid peroxidation and oxidative stress. On top of that, QUIN inhibits the uptake of glutamate into astrocytes by blocking glutamate transporters [35]; blockage of astrocytic glutamate uptake in the prefrontal cortex (PFC) induces complete cessation of responding in extreme cases [36], a need for higher minimum stimulation to maintain responding [36], impaired motor performance [36], diminished reward value and anhedonia [37], and cognitive impairment [37]; blockage of astrocytic glutamate uptake in the amygdala promotes reduction in social exploratory [38]. In addition, QUIN inhibits the expression of neurotrophins such as nerve growth factor (NGF) and brain-derived neurotrophic factor (BDNF) in a region-specific way [39], which has detrimental effects on the neurotrophins’ abilities to sustain the survival of neurons and prevent the negative outcome of QUIN and other KP metabolites. Low levels of neurotrophins were reliably associated with peripartum depression (e.g., [17]).

In the second branch of the KP, kynurenine is converted into kynurenic acid (KynA) by the kynurenine aminotransferase (KAT) enzymes (especially KAT-II [40]). Unlike the neurotoxicity of the former branch, KynA is generally considered neuroprotective [41], competitively inhibits ionotropic glutamate receptors at high concentrations, and attenuates activity at the glycine co-agonist site of the NMDAR [42]. As such, administering even low concentrations of KynA into the brain can decrease glutamate levels by 30–40% [43]. KynA, thus, works as car brakes and counters several of the adverse effects of the QUIN branch. Accordingly, a healthy balance must be maintained within the KP and the KP-serotonin conversion ratio to maintain sufficient serotonin and non-toxic levels of KP’s metabolites. Several factors can disrupt this delicate balance.

### The immune system and inflammation as balance disruptors

Inflammation is one key factor that might throw the conversion of tryptophan off homeostasis. During normal pregnancy, the immune system undergoes profound changes. Early in pregnancy, there is a pro-inflammatory stage that is crucial for implantation and placentation, characterized by increased production of cytokines, chemokines, and growth factors; next, comes an anti-inflammatory phase, characterized by rapid fetal growth; and lastly, another pro-inflammatory phase, preceding delivery in preparation for childbirth [44]. During the postpartum phase, the body gradually returns to nonpregnant homeostasis of immune regulation [45]. Pro-inflammatory cytokines direct the tryptophan metabolism towards kynurenine by upregulating IDO enzyme expression (mainly but not solely via the interferon-gamma receptor [46]) because activated immune cells need large amounts of energy to fight off infection, and hence QUIN is required to produce adequate amounts of NAD^+^. For example, during inflammation in the central nervous system (CNS), the concentration of QUIN in the brain homogenate and extracellular fluid increases by 246- and 66-fold, respectively [33], as a result of locally activated microglia. On top of that, macrophages in the bloodstream further increase the level of QUIN as they produce 20- to 30-fold more than microglia [33].

Therefore, when the immune system is over-exacerbated, repeatedly, or chronically expressed, this process might eventually result in neurotoxicity and serotonin depletion because it goes beyond the limits of homeostatic mechanisms such that the body can no longer sustain this state without negative effects [47]. A recent study found that inflammatory cytokines, particularly interleukin-6 (IL-6) and interleukin-1β (IL-1β), and KP metabolites, are associated with the severity of depressive symptoms in pregnancy and postpartum [23]. Therefore, individual differences in the immune system reactivity and/or IDO expression might account for part of the variance in PPD. There are, however, PPD episodes that are non-inflammatory in nature [48], which signifies that inflammation might be a powerful factor in PPD development but not a necessary one; a second key factor that might throw the conversion of tryptophan off homeostasis is the stress response.

### The stress response as a balance disruptor

Stress is common among pregnant women. From 2000 to 2010, data from the US-wide Centers for Disease Control Pregnancy Risk Assessment Monitoring System found that nearly 75% of postpartum mothers reported at least one major stressful event in the year leading up to the delivery of their baby [49]. The most cited stressors experienced during pregnancy comprised moving to a new address, arguing with a partner more than usual, severe illness and hospitalization of a family member, and inability to pay bills. These and other events may contribute to significant acute and chronic stress during pregnancy, and so to allostatic load [50] – load on the adaptive processes that maintain homeostasis through the production of mediators such as adrenalin, cortisol, and other chemical messengers that wear and tear on the body and brain. Stress steers the metabolism of tryptophan towards kynurenine, directly and indirectly; first, research has noted that the regulation of tryptophan dioxygenase (TDO)’s expression occurs mainly through glucocorticoid-receptor-mediated induction. Consequently, stress-related changes in the expression of TDO are primarily influenced by activation of the HPA-axis through the action of glucocorticoids such as cortisol [51]. Second, maternal stress is associated with cortisol release [52, 53]. High cortisol levels reduce lymphocyte sensitivity to glucocorticoids by binding to glucocorticoid receptors; subsequently, as steroid resistance is developed, there is an increased release of pro-inflammatory cytokines [52]. Maternal stress also influences circulating levels of inflammatory markers by increasing pro-inflammatory cytokines IL-1β, IL-6, and tumor necrosis factor α (TNF-α); an increase in pro-inflammatory factors upregulates the IDO enzyme expression that further intensifies kynurenine metabolism [25]. Third, glucocorticoid receptors also inhibit the function of important neurosteroids, such as 25-hydroxycholesterol (a cholestane) [54], which typically suppresses the activation of glutamate’s NMDA receptor [55]. By doing so, the stress response further intensifies the negative effects of QUIN on the NMDA receptor by blocking essential inhibitors of the process. Finally, chronic stress downregulates the expression of potassium-chloride cotransporter-2 (KCC2) [56], which causes the gamma-aminobutyric acid (GABA) receptors to exacerbate rather than inhibit the stress response.

Abundant research supports the association between stress and PPD: Childhood trauma, such as abuse and neglect, is associated with PPD in a dose-dependent manner and increases the likelihood of PPD even after adjusting for socioeconomic status, history of depression, and timing of onset [57]; Additionally, a meta-analysis of more than 14,000 cases indicated that stressful life events often precede the depressive episode [58]; Studies on PPD have highlighted several abnormalities in the activity of the HPA stress axis such as higher prenatal levels of cortisol and placental corticotropin-releasing hormone [59]; Finally, women with PPD had higher salivary evening cortisol levels six weeks postpartum as compared with healthy controls [60]. Therefore, individual differences in the sustained stress, reactivity, expression of the stress response, and TDO expression might account for additional variance in PPD.

### Women’s reproduction hormones as risk and protective factors

The third factor in UmPPD is the reproductive hormones, especially estrogen and its derivatives and progesterone (a neurosteroid), that might throw off the KP balance and interact with the immune system response. Estrogen and progesterone steadily increase during pregnancy (from an average of o.87 ng/mL in the 1st semester to 6.18 in the 3rd trimester and down to less than 0.1 postpartum for estrogen, and 17.48 to 70.45 and down to 0.86, respectively, for progesterone) and menstrual cycle (i.e., highest in the luteal phase [days 14–28]) [61], with considerable individual differences, particularly in estrogen levels [62]. Research has noted that estradiol disulfate and, to a weaker extent, estrone sulfate, estradiol, and estradiol 3-sulfate are all inhibitors of KAT enzymes [40], which dampen the expression of KynA and its neuroprotective effects, thereby intensifying the expression of the QUIN and NAD^+^ branch and its neurotoxic effects. Conversely, progesterone is a protective factor by acting as an activator of KynA [63] and an anti-inflammatory agent [64]; progesterone decreases the production of IL-1β, IL-6, TNFα, interferon γ (IFN-γ), and interleukin-12, as well as the production of chemokines [65]; during pregnancy, progesterone inhibits the production of pro-inflammatory cytokines and reduces the activity of T-helper type 1 cells (Th1) by altering chromatin accessibility in both promoter and distal regulatory structures associated to genes involved in T-cell activation, particularly the JUN transcription factor [66]. The regulation of T-cells is crucial as they play a critical role in suppressing autoimmune reactivity and the termination of the inflammatory response [67]. These anti-inflammatory effects are mediated by progesterone receptors (PRs) such that progesterone binds to PRs located in immune cells, including the natural killers, macrophages, dendritic cells, and T-cells, as well as in non-immune cells, such as epithelial and endothelial cells [68]. Prolonged and chronic inflammation, however, often causes progesterone resistance by reducing the affinity of the progesterone receptors (PR) either by epigenetic changes [69], alteration to steroid receptor chaperone proteins [70], or by direct competition for receptor coregulators [71]. Hence, prolonged inflammation indirectly disrupts the KP-serotonin balance by hindering the inhibitory nature of progesterone. Numerous studies link estrogen and progesterone with PPD. For example, in a recent study, higher estrogen and progesterone at two months postpartum were linked with more severe depressive symptoms over pregnancy. Estrogen was also positively associated with the pro-inflammatory cytokine IL-6 and negatively correlated with KynA. In contrast, progesterone was negatively correlated with IL-1β and several metabolites in the kynurenine pathway, including QUIN, kynurenine-tryptophan ratio, QUIN-picolinic acid ratio, and kynurenine [63]. Therefore, individual differences in the expression of estrogen and its derivatives and progesterone could account for another piece of the variance in PPD.

### Oxytocin as a protective factor against stress and inflammation

The fourth and final factor in the UmPPD is oxytocin. Oxytocin (OT) is a protective factor because it reduces inflammation [72] and the activity of the HPA stress axis [73] – the two main driving forces of the KP. The interplay of OT and the immune system is complex and multilayered [74, 75]; it is involved in the development of the immune system (e.g., stimulating bone mass and bone marrow [76]), immune surveillance and defense (e.g., plasma OT levels increase in early stages of sepsis [4–6 h], while decreasing in the brain, and decrease pro-inflammatory response [77]). Regarding stress, OT’s inhibition on the HPA stress axis is mainly conveyed via the GABA signaling pathway, particularly by potentiating the GABA_A_ receptor [78]. Accordingly, behaviors such as breastfeeding [79], skin-to-skin contact, and partner responsiveness [80] may promote the secretion of OT and its protective properties; lacking these behaviors may hinder the protective processes of OT and increase the likelihood of PPD and/or intensify PPD symptoms [81]. Likewise, in chronic stress, OT may increase stress because of the downregulation of KCC2 and the excitatory effect of GABA_A_. Finally, the KP is essential to the secretion of OT because NAD^+^ is the precursor for the CD38-cyclic ADP ribose (cADPR) pathway – a key mechanism in OT’s secretion [82]. CD38-cADPR is also essential to the recruitment, mobilization, and maintenance of the immune response [83]. Therefore, we hypothesize that in the early stages of inflammation, the production of NAD^+^ would result in the maintenance of the immune response and OT secretion via the CD38-cADPR pathway. In chronic inflammation, however, a between-tissue imbalance of CD38-cADPR may arise, decreasing the secretion of OT at the expense of intensifying the immune response. Therefore, individual differences in the expression of OT and behaviors associated with OT secretion could account for the final piece of the variance in PPD.

## PPD is not a perfect storm but a cluster of interconnected disorders

The proposed UmPPD theory can explain the full range of peripartum depression (PPD) symptoms. The excessive presence of QUIN – an agonist of glutamate’s NMDR receptor and blocker of the glutamate transporter – produces a glutamatergic surge in the brain, leading to excessive neurotoxicity, resulting in anhedonia, loss of energy, and increased fatigue, increased purposeless physical activity or slowed movements or speech, feelings of worthlessness or guilt, and lack of interest in the baby. Depleted serotonin can cause difficulty controlling mental pain, promoting thoughts of death and suicide, difficulty thinking, concentrating or making decisions, and changes in appetite. Depleted serotonin also causes a shortage of melatonin and disrupted sleep regulation.

The heterogeneity in PPD’s manifestation, likelihood, onset, and severity could be explained by the individual differences in the expression of UmPPD components. In that sense, PPD is not a perfect storm that only arises if multiple conditions are met, but a cluster of subtypes arising from a different composition of UmPPD components and a different expression of each element. For example, UmPPD predicts that depression could be inflammatory or noninflammatory because stress and sex hormones could create an imbalance in the KP and the KP-serotonin ratio regardless of inflammatory processes; it would predict, however, that the symptoms severity of inflammatory PPD would be more potent than noninflammatory PPD (see support in [84] regarding general depressive symptoms). Likewise, UmPPD predicts that PPD could be stress-induced or not stress-induced [85] because inflammation and sex hormones could create an imbalance in the KP and the KP-serotonin ratio regardless of stress. UmPPD would also predict that PPD is highly unlikely without stress or inflammation because these are the two driving forces behind the KP and the KP-serotonin ratio imbalance. On top of that, UmPPD accounts for the sexual dimorphism in the likelihood of PPD such that women are twice as likely to have PPD than men [86] and predicts that estrogen and its derivatives and progesterone are the two main forces behind this difference. UmPPD could also explain the mechanisms by which behaviors such as breastfeeding, rooming-in, and skin-to-skin contact decrease the likelihood of developing PPD [81] (i.e., via the protective processes of OT), and events such as unplanned cesarean section and delivery complications are linked with the probability and severity of PPD [87] (e.g., via the effects of stress on the imbalance in the KP and the KP-serotonin ratio). Finally, UmPPD may account for the findings that a history of depressive symptoms [88] and childhood trauma [57] increase the likelihood of developing PPD and relate to higher PPD severity. This latter ability of UmPPD is mediated by epigenetic modifications of its different components.

Epigenetics is the study of potentially heritable molecular alterations in DNA and histone proteins that can modify gene expression without changes in the underlying DNA sequence. DNA methylation, one of the most studied epigenetic processes, involves adding a methyl group to a cytosine nucleotide next to guanine in the DNA at a so-called CpG (cytosine-phosphate-guanine) site [89]. This modification can lead to gene silencing or stimulation of gene transcription, depending on where in the DNA it occurs [90]. DNA methylation is thought to be influenced by prenatal [91] and postnatal life events [92] and its state is reversible [93]. It can therefore be seen as the dynamic interface between genes and the environment [94], and its function can be compared to a dimmer switch that regulates gene expression based on the degree of methylation density. As such, DNA methylation may account for some of the individual differences in the expression of genes and subsequently in the functioning of various systems, such as the immune system, stress response, reproductive hormones, OT, and KP. Childhood trauma, as an exemplar, may promote specific regulatory changes in HPA stress axis genes [95] and inflammatory-related genes [96] that would later in life increase the stress and/or immune system reactivity and more easily throw off balance the KP and the KP-serotonin ratio.

## Application of the UmPPD to basic and clinical research

The search for an effective treatment for depression symptoms dates to the second millennium in Babylon. Despite centuries of research, a truly effective remedy for depression still eludes us. One of the first drugs developed was Imipramine, a tricyclic antidepressant [97], followed by Prozac (fluoxetine), a selective serotonin reuptake inhibitor (SSRI) [97]. These drugs work by preventing the reabsorption of serotonin in the synaptic cleft. The UmPPD suggests that the limited effectiveness of these drugs may be due to the hyperactivation of the kynurenine pathway (KP), which leads to the depletion of serotonin through the function of the IDO and TDO enzymes. While tricyclic and SSRI antidepressants may alleviate some of the symptoms of depression by preventing the complete depletion of serotonin, they do not address the main cause of depression, which is the uncontrolled metabolites of the KP, especially quinolinic acid (QUIN). A second line of drugs for PPD, such as the recently developed and FDA-approved Brexanolone [98], targets the GABA_A_ receptors. According to UmPPD, these drugs can reduce the uncontrolled KP metabolites by inhibiting stress and immune responses through the GABAergic system. However, these drugs are not expected to alleviate the toxic KP metabolites that are already present and may only provide temporary relief, as they merely slow down the driving forces by acting on the brakes of the GABAergic system. They may also backfire if the KCC2 is downregulated. A third promising line of drugs targets the NMDA receptor of glutamate, such as ketamine [99]. The UmPPD theory suggests that these drugs could provide quick relief for most depression symptoms by negating the effects of QUIN on the glutamate system and increasing the number of postsynaptic NMDARs. However, while these drugs may alleviate most depression symptoms, they do not address the driving forces behind the KP, and thus their effects may be short-lived. According to UmPPD, the best approach to treating depression would be to target the IDO and TDO enzymes directly, using drugs such as 1-methyl-D-tryptophan and allopurinol, respectively. This would promptly reduce the toxic levels of the KP metabolites. The most effective drug cocktail would consist of IDO and TDO inhibitors and ketamine, or brexanolone and ketamine, as these combinations target both the driving forces of the KP and the toxic KP metabolites that are already present.

Regarding psychological intervention of PPD, research indicates moderate and highly varied effectiveness of cognitive behavior therapies (g_mean_ = 0.64) and interpersonal psychotherapy (g_mean_ = 0.53) schedules, with higher success for group formats [11]. One core component that predicts therapeutic success is the working alliance because it relates to engagement with the process, fewer barriers, and greater effectiveness [100]. UmPPD suggests that one aspect that could account for the relative success of psychological interventions is the activation/reactivation of the oxytocin system via the strong working alliance, which reduces the activation of the stress and immune responses and decreases the toxicity of the KP metabolites.

## Closing remarks

UmPPD attempts to unify state-of-the-art knowledge on PPD in one cohesive network of dependent mechanisms in which the KP is the central hub. UmPPD predicts a higher likelihood of developing PPD, early onset, and greater symptoms severity would be associated with prolonged inflammation, ongoing or multiple stressors, excessive estrogen, progesterone resistance, avoidance of breastfeeding, skin-to-skin contact, and social proximity. Subtypes of PPD would consist of different compositions and expressions of these components, with one central common factor – the imbalance within the KP and the KP-serotonin ratio. UmPPD could aid in directing future research and possibly detecting critical processes that could help discover, develop, and utilize novel treatments for PPD.
